# Additional assessment of Acute Cystitis Symptom Score questionnaire for patient-reported outcome measure in female patients with acute uncomplicated cystitis: part II

**DOI:** 10.1007/s00345-019-02948-8

**Published:** 2019-09-23

**Authors:** J. F. Alidjanov, K. G. Naber, A. Pilatz, A. Radzhabov, M. Zamuddinov, A. Magyar, P. Tenke, F. M. Wagenlehner

**Affiliations:** 1 Clinic for Urology, Pediatric Urology and Andrology, Justus Liebig University, Giessen, Germany; 2 Department of Urology, Technical University of Munich, Munich, Germany; 3 Treatment and Diagnostic Center “Olami Tib”, Dushanbe, Tajikistan; 4 Sankt-Katharinen Hospital, Frankfurt, Germany; 5 Department of Urology, Madadi Akbar Clinic, Dushanbe, Tajikistan; 6 Department of Urology, Jahn Ferenc South Pest Teaching Hospital, Budapest, Hungary

**Keywords:** Urinary tract infection, Cystitis, Acute cystitis symptom score, ACSS, Guidelines, Patient-reported outcome

## Abstract

**Purpose:**

Since symptomatic, non-antibiotic therapy has become an alternative approach to treat acute cystitis (AC) in women, suitable patient-reported outcome measures (PROM) are urgently needed. The aim of this part II of a larger non-interventional, case–control study was the additional assessment of the ACSS as a suitable PROM.

**Methods:**

Data from 134 female patients with diagnosed acute uncomplicated cystitis were included in the current analysis with (1) a summary score of “Typical” domain of 6 and more; (2) at least one follow-up evaluation after the baseline visit; (3) no missing values in the ACSS questionnaire data. Six different predefined thresholds based on the scoring of the ACSS items were evaluated to define “clinical cure”, also considering the draft FDA and EMA guidelines.

**Results:**

Of the six different thresholds tested, a summary score of the five typical symptoms of 5 and lower with no symptom more than 1 (mild), without visible blood in urine, with or without including QoL issues was favoured, which partially also could be adapted to the draft FDA and EMA guidelines. The overall patient’s clinical assessment (“Dynamic” domain) alone was not sensitive enough for a suitable PROM.

**Conclusions:**

Scoring of the severity of symptoms is needed not only for diagnosis, but also for PROM to define “clinical cure” of any intervention, which could be combined with QoL issues. Results of the study demonstrated that the ACSS questionnaire has the potential to be used as a suitable PROM and should further be tested in prospective clinical studies.

**Electronic supplementary material:**

The online version of this article (10.1007/s00345-019-02948-8) contains supplementary material, which is available to authorized users.

## Introduction

Although current guidelines recommend the use of antibiotics (ABs) as the first choice of treatment for the acute phase of uncomplicated urinary tract infections (uUTI) [1, 2], several prospective randomized, controlled studies have been performed already comparing antibiotic therapy with symptomatic therapy of uncomplicated acute cystitis (AC) in women [3–6]. These results were compelling enough for the updated German Clinical Guidelines [2] to encourage the use of non-AB symptomatic treatment in selected cases of acute lower uUTIs with mild-to-moderate symptoms. Taking into account the possible protective abilities of asymptomatic bacteriuria against recurrent UTI, it has become obvious that the elimination of bacteriuria cannot be considered anymore the main aim of studies focused on the assessment of the efficacy of non-antibiotic modalities in the treatment of AC [7, 8]. Consequently, suitable and effective patient-reported outcome measures (PROM) are urgently needed.

According to the Food and Drug Administration (FDA) guidance for industry, a PROM is “a means to capture PROM data used to measure treatment benefit or risk in medical product clinical trials”. Additional definition of a PROM includes the following: “any report of the status of a patient’s health condition that comes directly from the patient, without interpretation of the patient’s response by a clinician or anyone else. The outcome can be measured in absolute terms (e.g., the severity of a symptom, sign, or state of a disease) or as a change from a previous measure. In clinical trials, a PROM can be used to measure the effect of a medical intervention on one or more concepts (i.e., the *thing* being measured, such as a symptom or group of symptoms, effects on a particular function or group of functions, or a group of symptoms or functions shown to measure the severity of a health condition)” [9].

The Acute Cystitis Symptom Score (ACSS) was already introduced as a standardized self-reporting diagnostic questionnaire, which has proven its efficacy in the clinical diagnosis of AC in women and in monitoring possible changes after therapy [10–14]. The ACSS has been translated and validated in several languages and is available online (http://www.acss.world/downloads.html). In a smaller, non-interventional study, the ACSS was already evaluated as a PROM [13, 14]. Since the ACSS has now been used in a larger non-interventional, case–control study [15], we aimed to perform an additional assessment of the ACSS as a suitable PROM.

## Materials and methods

### Study design

The current study was planned as a non-interventional within-subject design and can be considered as part II of the recent publication [15], which mainly analysed the diagnostic values of the ACSS as compared to the recently published draft guidelines of FDA and EMA [16, 17].

### Study tool

The ACSS is composed of the “Diagnostic” and “Follow-up” forms (part A and part B). Each of these forms consists of four domains: (1) typical symptoms, (2) differential symptoms, (3) quality of life (QoL), (4) additional medical conditions. Besides the four mentioned domains, the “Follow-up” part B of the ACSS contains the “Dynamics” domain to assess the overall clinical outcome reported by the patient [10].

The “Typical” domain of the ACSS contains six patient-reported items corresponding to (1) urination frequency, (2) urination urgency, (3) burning pain during urination (dysuria), (4) suprapubic pain, (5) incomplete bladder emptying, vi) visible blood in the urine.

The “QoL” domain is composed of three items concerning (1) overall discomfort (bothersomeness) caused by the symptoms and their severity, (2) impact on daily work/activities, and (3) impact on social activities.

The items of the “Typical” and “QoL” domains were scored according to severity: none, mild, moderate, and severe.

The “Differential” domain of the ACSS contains items concerning differential diagnostic considerations, such as female genital infections and upper UTI symptoms. The “Additional” domain contains questions concerning important medical conditions, such as menstruation, premenstrual syndrome (PMS), postmenopause, pregnancy, and diabetes mellitus.

The “Dynamics” domain of the ACSS is composed of five grades concerning overall changes of the symptomatology: Feeling (1) normal (all symptoms have gone away); (2) much better (most of the symptoms has gone away); (3) somewhat better (only some symptoms have gone away); (4) no changes; (5) worse.

The data from both “Diagnostic” and “Follow-up” forms of the ACSS questionnaire were used in this study.

From the draft guidelines proposed by FDA, the four (dysuria, urinary frequency, urinary urgency, and suprapubic pain) or by EMA, the three (frequency, urgency and dysuria) symptoms mentioned in the corresponding draft guidelines—all included also in the ACSS questionnaire—were analysed accordingly [16, 17]. All items were dichotomized (s. below) as “Positive” or “Negative”, depending on the presence or absence of the symptom, and their severity was also considered.

### Data acquisition

The e-USQOLAT database, containing relevant clinical information and laboratory data of women with and without AC was selected as a primary source for data mining [18]. These data were obtained from female respondents at baseline and follow-up visits during clinical validation of the ACSS in several countries. All relevant data were acquired from the database at its state on the access date of January 1, 2019.

### Data processing

**Fig. 1:**
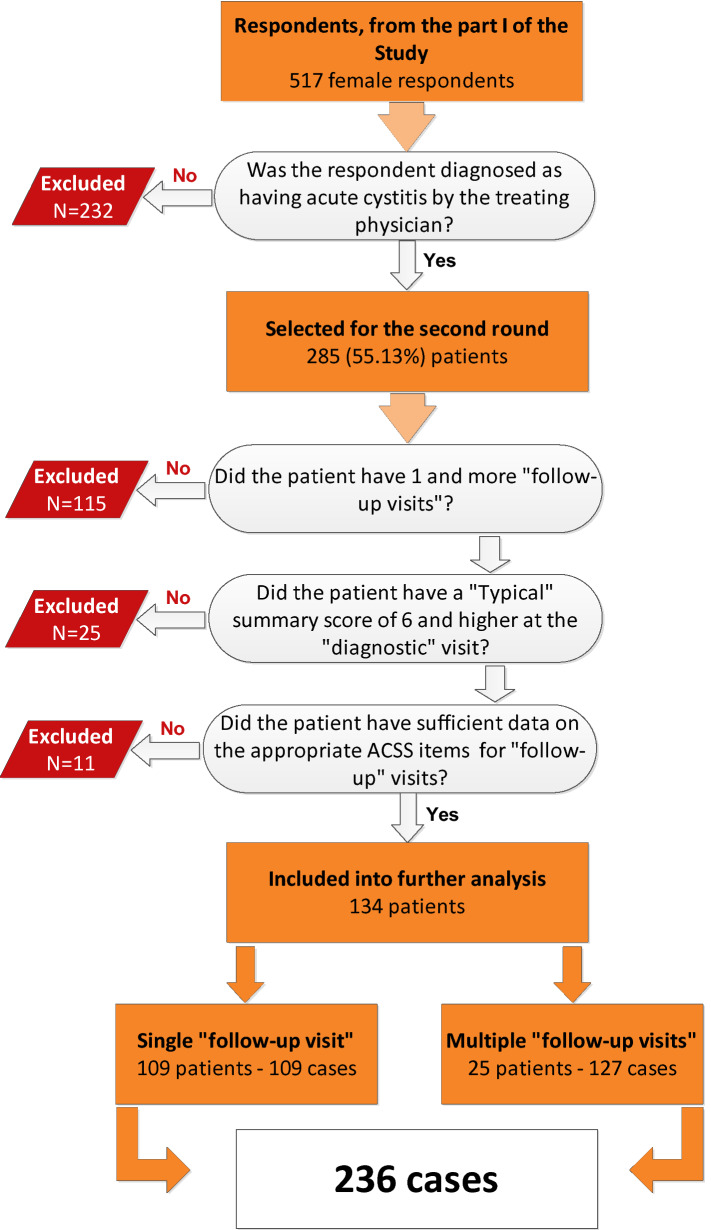
Flowchart of patients’ selection. Part I of the study [15]

Of among 517 female respondents, described in our recent publication [15], we have selected patients with AC according to the diagnosis made by the treating physician with the following inclusion criteria: (1) summary score of “typical symptoms” of 6 and more; (2) at least one follow-up evaluation after the initial “diagnostic” visit; (3) no missing values in the ACSS questionnaire data, including the “Dynamics” domain of the “follow-up Part B” of the questionnaire (Fig. 1).

Patients were supposed to receive appropriate medical treatment according to the national and international guidelines and therapeutic standards [1, 2, 19]. However, only outcome and not therapy modalities were included in the further analysis of this non-interventional study.

Patients, who have filled up more than 1 “follow-up Part B” of the ACSS were added as new cases per each available follow-up form (visit). Visits were grouped depending on the time difference between the first diagnostic visit and further “follow-up” evaluation visits.

The “Dynamics” domain of the “follow-up Part B” form of the ACSS was also considered for evaluation of overall clinical outcome determined by the patient. In the purpose of dichotomization, items “Yes I feel normal” and “Yes, I feel much better” were merged and classified as “clinical cure”, whereas the three remaining items (“Yes, I feel somewhat better”, “No, there are barely any changes”, and “Yes, I feel worse”) were merged to “failure”. The procedures of dichotomization were described previously [15].

In general, relative variables were labelled as “0” for “negative,”/“not match”, and “1” for “positive”/“match”.

### Thresholds and terms

The evaluation terms or “visits” were classified according to the time difference (in days) between the “diagnostic” and “follow-up” evaluations.

To determine meaningful thresholds for clinical cure, typical symptoms, QoL and overall clinical assessments (“Dynamic” domain) were evaluated, combined and/or weighed against each other.

### Statistical analysis

Two-by-two contingency tables were used for the statistical analysis of the bivariate (dichotomized) variables, where the thresholds in different times of the evaluation were considered as the test variable (exposure), and efficacy of the therapy was taken as an outcome.

The validity of the predetermined thresholds was evaluated by the assessment of their relations with the overall clinical outcome as reported by the patients in the “Dynamics” domain of the “follow-up” form of the ACSS.

Such values as sensitivity, specificity, positive and likelihood ratios, Youden’s J-index, odds ratio (OR), positive and negative predictive values (PPV and NPV respectively), positive and negative likelihood ratios (+LR and −LR respectively) were calculated. ROC-curve analysis was used for the assessment of area under the curve (AUC). The strength of associations between test variables and the outcome was measured using Pearson’s product–moment correlation coefficient.

Tests of the comparative analyses were performed in dependence of normality and homoscedasticity of distributions which in turn were assessed using normality tests (Shapiro–Wilk’s) [20], histograms and normal Q–Q plots (see Suppl. Figures 1 and 2).

For the comparison of independent, homoscedastic and normally distributed variables, Student’s two-sided *t* test was used. For normally distributed heteroscedastic independent variables, Welch’s two-sided modified *t* test was used. Non-parametric tests such as Kruskal–Wallis rank-sum test [21] and Wilcoxon/Mann–Whitney rank-sum test for pairwise comparisons [22] were used when parametric tests were considered inappropriate. A *p* value of less than 0.05 was considered statistically significant.

R v.3.5.2 with in-built and additional packages was used for the statistical analysis and graphical representation of the results [23–26].

## Results

Using the criteria described above, 134 patients of among 517 previously selected female respondents [15] were included in the current analysis. The age of the selected patients ranged from 17 to 82 years, with a median (IQR) of 31 (24.00–44.25) and mean (SD) of 36.28 (16.03) years. Of them, 109 filled up at least 1 copy of the “follow-up Part B” form of the ACSS (one “follow-up” visit) after the initial “diagnostic” visit and 25 patients filled up multiple copies at different “follow-up” visits. Altogether, they have formed 236 cases (Fig. 1).

The maximum time difference between “diagnostic” (visit 1) and “follow-up” evaluations (FU visits) was 29 days. According to the time difference, we have classified four terms of the “follow-up” evaluations: (1) Very early evaluation or “Visit 2” (less than 2 days between “diagnostic” and “follow-up” evaluations); (2) Early evaluation or “Visit 3” (2–4 days between “diagnostic” and “follow-up” evaluations); (3) End-of-therapy evaluation or “Visit 4” (5–9 days between “diagnostic” and “follow-up” evaluations), and (4) Test-of-cure evaluation or “Visit 5” (10–30 days between “diagnostic” and “follow-up” evaluations).

Eight different thresholds for evaluation of clinical cure at the outcome were predetermined:

- A. A summary score of the “Typical” domain up to 5 AND no visible blood in the urine
- B. A summary score of the “Typical” domain up to 4 AND no visible blood in the urine
- C. A summary score of the “Typical” domain up to 5 with no item > 1 (mild) AND no visible blood in the urine
- D. A summary score of the “Typical” domain up to 4 AND no “Typical” item > 1 (mild) AND no visible blood in the urine
- E. A summary score of the “Typical” domain up to 5 AND no “Typical” item > 1 (mild) AND no visible blood in the urine AND no “QoL” item > 1
- F. A summary score of the “Typical” domain up to 4 AND no “Typical” item > 1 AND no visible blood in the urine AND no “QoL” item > 1
- G. A summary score of the four FDA symptoms up to 4 AND no score > 1 (mild) AND no visible blood in the urine
- H. A summary score of the three EMA symptoms up to 3 AND no score > 1 (mild) AND no visible blood in the urine

Six of these thresholds (A–F) are related to the ACSS items, one (G) was adapted to the FDA criteria, considering four symptoms, and one (H) was adapted to the EMA criteria, considering three symptoms, as suggested in the corresponding draft guidelines [16, 17].

Since only 34.75% of patients had visible blood in urine, which decreased to only two patients at visits 4 and 5, we considered a clinical cure for all of the thresholds only for cases with no visible blood as stated by the patient.

**Fig. 2:**
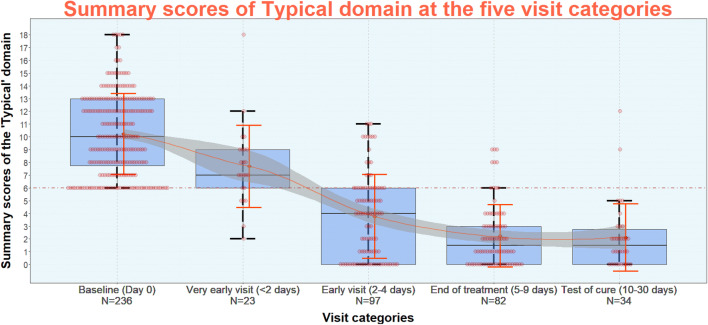
Summary scores of “Typical” domain of ACSS at diagnostics of acute uncomplicated cystitis (AC) in women (baseline) and at the four different follow-up visit categories: “very early visit”, “early visit”, “end-of-treatment visit”, “test-of-cure visit”. *Note* Red dots represent cases, orange diamonds represent mean scores, orange error bars represent standard deviations, orange line illustrates the symptomatic “course” of AC, grey “strip” around the orange line represents standard error of a mean

At the time of “diagnostic” evaluation (visit 1), median (IQR) of the summary typical score by the patients was 10 (7.75–13.00). On the next day of therapy (very early evaluation/visit 2), it reduced to 7.00 (6.00–9.00). Further reductions were as follows: 4.00 (0.00–6.00) at the early evaluation (visit 3), 1.50 (0.00–3.00) at the end-of-therapy evaluation (visit 4), and 1.50 (0.00–2.75) at the test-of-cure evaluation (visit 5). The average summary scores of the “Typical” domain differed significantly between all evaluation categories (*p* < 0.05), except between those at end-of-therapy and test-of-cure evaluations (*p* = 0.71) (Table 1, Fig. 2).

**Table 1:** Summary scores of “Typical” domain at the five visit categories (mean, SD, median, IQR)

	Cases (*n*)	Sum score of typical domain				
		Mean	SD	Median	IQR	
Visit 1 (diagnostics, Day 0)	236	10.23	3.18	10.00	7.75	13.00
Visit 2 (very early FU, Day < 2)	23	7.70	3.21	7.00	6.00	9.00
Visit 3 (early FU, Day 2–4)	97	3.77	3.29	4.00	0.00	6.00
Visit 4 (end of treatment, Day 5–9)	82	2.26	2.94	1.50	0.00	3.00
Visit 5 (test of cure, Day 10–30)	34	2.12	3.38	1.50	0.00	2.75

The severity of the six typical symptoms at visit 1 and the follow-up visits are presented in Table 2. At the “diagnostic” visit 1, five of six typical symptoms were positive in 88.98–97.03% of the cases. Although the percentage of cases with positive symptoms decreased over the observation time (especially starting from visit 3), and the percentages of cases with severe or moderate symptoms decreased significantly, a relatively high proportion of cases of at least mild symptoms remained even up to visit 5. Visible blood in the urine (a pathognomonic symptom of hemorrhagic cystitis) was found only in 34.75% of cases at the “diagnostic” visit 1 and was reduced to only two cases at the visits 4 (2.44%) and 5 (5.88%).

**Table 2:** Typical symptoms and their severity claimed by the patients at the five visit categories

Visit 1	Diagnostics (Day 0). *n* of cases = 236			
Typical symptoms	Total (%)	Mild (%)	Moderate (%)	Severe (%)
Urinary frequency	210 (88.98%)	69 (29.24%)	69 (29.24%)	72 (30.51%)
Urinary urgency	220 (93.22%)	40 (16.95%)	88 (37.29%)	92 (38.98%)
Dysuria	229 (97.03%)	38 (16.10%)	72 (30.51%)	119 (50.42%)
Suprapubic pain	196 (83.05%)	62 (26.27%)	83 (35.17%)	51 (21.61%)
Incomplete bladder emptying	218 (92.37%)	62 (26.27%)	89 (37.71%)	67 (28.39%)
Visible blood in urine	82 (34.75%)	43 (18.22%)	21 (8.90%)	18 (7.63%)

Visit 2	Very early FU visit (in less than 2 days). *n* of cases = 23			
Typical symptoms	Total (%)	Mild (%)	Moderate (%)	Severe (%)
Urinary frequency	18 (78.26%)	10 (43.48%)	5 (21.74%)	3 (13.04%)
Urinary urgency	22 (95.65%)	13 (56.52%)	8 (34.78%)	1 (4.35%)
Dysuria	22 (95.65%)	5 (21.74%)	12 (52.17%)	5 (21.74%)
Suprapubic pain	18 (78.26%)	8 (34.78%)	6 (26.09%)	4 (17.39%)
Incomplete bladder emptying	19 (82.61%)	8 (34.78%)	8 (34.78%)	3 (13.04%)
Visible blood in urine	4 (27.39%)	2 (8.70%)	1 (4.35%)	1 (4.35%)

Visit 3	Early FU visit (2–4 days). *n* of cases = 97			
Typical symptoms	Total (%)	Mild (%)	Moderate (%)	Severe (%)
Urinary frequency	42 (43.30%)	28 (28.87%)	12 (12.37%)	2 (2.06%)
Urinary urgency	51 (52.58%)	36 (37.11%)	13 (13.40%)	2 (2.06%)
Dysuria	59 (60.82%)	40 (41.24%)	14 (14.43%)	5 (5.15%)
Suprapubic pain	49 (50.52%)	32 (32.99%)	10 (10.31%)	7 (7.22%)
Incomplete bladder emptying	50 (51.55%)	38 (39.18%)	9 (9.28%)	3 (3.09%)
Visible blood in urine	13 (13.40%)	8 (8.25%)	4 (4.12%)	1 (1.03%)

Visit 4	End-of-treatment FU visit (5–9 days). *n* of cases = 82			
Typical symptoms	Total (%)	Mild (%)	Moderate (%)	Severe (%)
Urinary frequency	24 (29.27%)	16 (19.51%)	7 (8.54%)	1 (1.22%)
Urinary urgency	33 (40.24%)	26 (31.71%)	6 (7.32%)	1 (1.22%)
Dysuria	37 (45.12%)	33 (40.24%)	3 (3.66%)	1 (1.22%)
Suprapubic pain	24 (29.27%)	19 (23.17%)	3 (3.66%)	2 (2.44%)
Incomplete bladder emptying	28 (34.15%)	21 (25.61%)	7 (8.54%)	0 (0.00%)
Visible blood in urine	2 (2.44%)	1 (1.22%)	1 (1.22%)	0 (0.00%)

Visit 5	Test-of-cure visit (10–30 days). *n* of cases = 34			
Typical symptoms	Total (%)	Mild (%)	Moderate (%)	Severe (%)
Urinary frequency	14 (41.28%)	12 (35.29%)	1 (2.94%)	1 (2.94%)
Urinary urgency	14 (41.28%)	12 (35.29%)	1 (2.94%)	1 (2.94%)
Dysuria	15 (44.12%)	12 (35.29%)	3 (8.82%)	0 (0.00%)
Suprapubic pain	6 (17.65%)	5 (14.71%)	1 (2.94%)	0 (0.00%)
Incomplete bladder emptying	8 (23.53%)	6 (17.65%)	1 (2.94%)	1 (2.94%)
Visible blood in urine	2 (5.88%)	2 (5.88%)	0 (0.00%)	0 (0.00%)

Table 3 represents the results of the assessment of the quality of life (QoL). It can be seen that the symptoms of acute cystitis affect all three indicated categories of QoL in almost all the cases (96.6–98.7%). Although the higher rates of severity (moderate, severe) were reduced during follow-up, about one-third of patients still claimed at the least mild impact on their QoL in all three categories.

**Table 3:** Impact on quality of live at the five visit categories

Visit 1	Diagnostic visit (Day 0). *n* of cases = 236			
Impact on quality of life	Total (%)	Mild (%)	Moderate (%)	Severe (%)
Overall discomfort	233 (98.73%)	38 (16.10%)	135 (57.20%)	60 (25.42%)
Work/daily activities	230 (97.46%)	86 (36.44%)	111 (47.03%)	33 (13.98%)
Social activities	228 (96.61%)	100 (42.37%)	89 (37.71%)	39 (16.53%)

Visit 2	Very early FU visit (in less than 2 days). *n* of cases = 23			
Impact on quality of life	Total (%)	Mild (%)	Moderate (%)	Severe (%)
Overall discomfort	18 (78.26%)	10 (43.48%)	5 (21.74%)	3 (13.04%)
Work/daily activities	22 (95.65%)	13 (56.52%)	8 (34.78%)	1 (4.35%)
Social activities	22 (95.65%)	5 (21.74%)	12 (52.17%)	5 (21.74%)

Visit 3	Early FU visit (2–4 days). *n* of cases = 97			
Impact on quality of life	Total (%)	Mild (%)	Moderate (%)	Severe (%)
Overall discomfort	42 (43.30%)	28 (28.87%)	12 (12.37%)	2 (2.06%)
Work/daily activities	51 (52.58%)	36 (37.11%)	13 (13.40%)	2 (2.06%)
Social activities	59 (60.82%)	40 (41.24%)	14 (14.43%)	5 (5.15%)

Visit 4	End-of-treatment FU visit (5–9 days). *n* of cases = 82			
Impact on quality of life	Total (%)	Mild (%)	Moderate (%)	Severe (%)
Overall discomfort	24 (29.27%)	16 (19.51%)	7 (8.54%)	1 (1.22%)
Work/daily activities	33 (40.24%)	26 (31.71%)	6 (7.32%)	1 (1.22%)
Social activities	37 (45.12%)	33 (40.24%)	3 (3.66%)	1 (1.22%)

Visit 5	Test of cure (10–30 days). *n* of cases = 34			
Impact on quality of life	Total (%)	Mild (%)	Moderate (%)	Severe (%)
Overall discomfort	14 (41.18%)	12 (35.29%)	1 (2.94%)	1 (2.94%)
Work/daily activities	14 (41.18%)	12 (35.29%)	1 (2.94%)	1 (2.94%)
Social activities	15 (44.12%)	12 (35.29%)	3 (8.82%)	0 (0.00%)

The percentage of cases with “back to normal” or “much better” in the “Dynamics” domain have increased over the follow-up time, but there was still a noticeable number of the cases stated as “somewhat better” (Table 4). Therefore, it is difficult to decide how “clinical cure” should be defined in the frame of the current study using only the “Dynamics” domain by itself.

**Table 4:** Overall changes (ACSS “Dynamics”) from visit 1 at the four follow-up visit categories

ACSS (Dynamics) *n* of cases	Feeling normal (*n*, %)	Much better (*n*, %)	Somewhat better (*n*, %)	No changes (*n*, %)	Feeling worse (*n*, %)
Visit 2 (Day < 2), *n* = 23	0 (0.00%)	1 (4.35%)	12 (52.17%)	9 (39.13%)	1 (4.35%)
Visit 3 (Day 2–4), *n* = 97	17 (17.53%)	39 (40.21%)	31 (31.96%)	9 (9.28%)	1 (1.03%)
Visit 4 (Day 5–9), *n* = 82	24 (29.27%)	40 (48.78%)	12 (14.63%)	3 (3.66)	3 (3.66%)
Visit 5 (Day 10–30), *n* = 34	14 (41.18%)	10 (29.41%)	10 (29.41%)	0 (0.00%)	0 (0.00%)

Visit 2 (very early, Day < 2); visit 3 (early, Day 2–4); visit 4 (end of treatment, Day 5–9); visit 5 (test of cure, Day 10–30)

In Table 5, the results of the Tables 2–4 are summarized using for the six items of the “Typical” domain and the three items of the “QoL” domain the percentages of cases rating their symptoms and impact on QoL as moderate or severe at visit 1 (diagnostics) and the three follow-up visits (early, end of treatment, test of cure) and the patient’s overall clinical assessment (“Dynamics” domain) according two different thresholds at the same three follow-up visits. It can be seen that the scoring of the symptoms (except visible blood in urine) and the “QoL” items are decreasing fairly parallel starting from visit 1 to visit 5. As mentioned above, establishing a threshold between “feeling much better” and “feeling somewhat better” would show far too low “clinical cure” rates which are not compatible with clinical experience in patients with AC.

**Table 5:** Percentage of cases rating their symptoms and impact on the quality-of-life parameters as moderate and severe at visit 1 (diagnostics) and at three follow-up visits (early, end of treatment, a test of cure) and the patient’s overall assessment (Dynamics domain) according to two different thresholds at the same three follow-up visits

	Visit 1 (*n* = 236)	Visit 3 (*n* = 97)	Visit 4 (*n* = 82)	Visit 5 (*n* = 34)
Typical symptoms (moderate + severe)				
Urinary frequency	59.75%	14.43%	9.76%	5.88%
Urinary urgency	76.27%	15.46%	8.54%	5.88%
Dysuria	80.93%	19.58%	4.88%	8.82%
Suprapubic pain	86.78%	17.53%	6.10%	2.94%
Incomplete bladder emptying	66.10%	12.37%	8.54%	5.88%
Visible blood in urine	16.53%	4.12%	1.22%	0%
Quality of life (moderate-to-severe impact)				
Overall discomfort	82.62%	14.43%	9.76%	5.98%
Work/daily activities	70.01%	15.46%	8.54%	5.98%
Social activities	54.34%	19.58%	4.88%	0%
Dynamics				
“Somewhat better, no changes, feeling worse”		50.27%	21.95%	29.41%
“No changes, feeling worse”		10.31%	7.32%	0%

Finally, the results of the eight different predetermined thresholds—six related to ACSS items and one adapted each to FDA and EMA criteria—analysed at the different follow-up visits concerning discrimination of clinical cure depending on the answers of the patients are shown in Table 6. In general, the results demonstrate again that using severity of symptoms combined with or without QoL items fairly comparable rates of “clinical cure” could be obtained.

**Table 6:** A number of cases above and below certain breakpoints representing success and non-success at the four follow-up visit categories. Each case with “visible blood in the urine (VBU)” was rated “non-success”. (Threshold letters adjusted to supplementary table 1)

Criteria for success and non-success	Yes (*n*, %)	No (*n*, %)
Visit 2 (very early, Day < 2), *n* of cases = 23		
A) summary score of typical domain ≤ 5 scores and “visible blood in urine” = 0	5 (21.74%)	18 (78.26%)
B) summary score of typical domain ≤ 4 scores and “visible blood in urine” = 0	2 (8.70%)	21 (91.3%)
C) summary score of typical domain ≤ 5 scores, no item > 1 and “visible blood in urine” = 0	2 (8.70%)	21 (91.3%)
D) summary score of typical domain ≤ 4 scores, no item > 1 and “visible blood in urine” = 0	2 (8.70%)	21 (91.3%)
E) summary score of typical domain < 5 scores, no item > 1 and no item of QoL > 1 and “visible blood in urine” = 0	1 (4.35%)	22 (95.65%)
F) summary score of typical domain ≤ 4 scores, no item > 1 and no item of QoL > 1 and “visible blood in urine” = 0	1 (4.35%)	22 (95.65%)
G) summary score of 4 FDA symptoms ≤ 4, no item > 1 and “visible blood in urine” = 0	2 (8.70%)	21 (91.3%)
H) summary score of 3 EMA symptoms ≤ 3, no item> 1 and “visible blood in urine” = 0	2 (8.70%)	21 (91.3%)
Visit 3 (early, Day 2–4), *n* of cases = 97		
A) summary score of typical domain ≤ 5 scores and “visible blood in urine” = 0	64 (65.98%)	33 (34.02%)
B) summary score of typical domain < 4 scores and “visible blood in urine” = 0	54 (55.67%)	43 (44.33%)
C) summary score of typical domain ≤ 5 scores, no item > 1 and “visible blood in urine” = 0	55 (56.70%)	42 (43.30%)
D) summary score of typical domain ≤ 4 scores, no item > 1 and “visible blood in urine” = 0	51 (52.58%)	46 (17.42%)
E) summary score of typical domain ≤ 5 scores, no item > 1 and no item of QoL > 1 and “visible blood in urine” = 0	53 (54.64%)	44 (45.36%)
F) summary score of typical domain ≤ 4 scores, no item > 1 and no item of QoL > 1 and “visible blood in urine” = 0	50 (51.55%)	47 (48.45%)
G) summary score of 4 FDA symptoms ≤ 4, no item > 1 and “visible blood in urine” = 0	56 (57.73%)	41 (42.27%)
H) summary score of 3 EMA symptoms ≤ 3, no item > 1 and “visible blood in urine” = 0	59 (60.82%)	38 (39.18%)
Visit 4 (end of treatment, Day 5–9), *n* of cases = 82		
A) summary score of typical domain ≤ 5 scores and “visible blood in urine” = 0	70 (85.37%)	12 (14.63%)
B) summary score of typical domain ≤ 4 scores and “visible blood in urine” = 0	69 (84.15%)	13 (15.85%)
C) summary score of typical domain ≤ 5 scores, no item > 1 and “visible blood in urine” = 0	66 (80.49%)	16 (19.51%)
D) summary score of typical domain ≤ 4 scores, no item > 1 and “visible blood in urine” = 0	66 (80.49%)	16 (19.51%)
E) summary score of typical domain ≤ 5 scores, no item > 1 and no item of QoL > 1 and “visible blood in urine” = 0	60 (73.17%)	22 (26.83%)
F) summary score of typical domain ≤ 4 scores, no item > 1 and no item of QoL > 1 and “visible blood in urine” = 0	60 (73.17%)	22 (26.83%)
G) summary score of 4 FDA symptoms ≤ 4, no item > 1 and “visible blood in urine” = 0	66 (80.49%)	16 (19.51%)
H) summary score of 3 EMA symptoms ≤ 3, no item > 1 and “visible blood in urine” = 0	67 (81.71%)	15 (18.28%)
Visit 5 (test of cure, Day 10–30), *n* of cases = 34		
A) summary score of typical domain ≤ 5 scores and “visible blood in urine” = 0	30 (88.24%)	4 (11.76%)
B) summary score of typical domain ≤ 4 scores and “visible blood in urine” = 0	28 (82.35%)	6 (17.65%)
C) summary score of typical domain ≤ 5 scores, no item > 1 and “visible blood in urine” = 0	28 (82.35%)	6 (17.65%)
D) summary score of typical domain ≤ 4 scores, no item > 1 and “visible blood in urine” = 0	27 (79.41%)	7 (20.59%)
E) summary score of typical domain ≤ 5 scores, no item > 1 and no item of QoL > 1 and “visible blood in urine” = 0	27 (79.41%)	7 (20.59%)
F) summary score of typical domain ≤ 4 scores, no item > 1 and no item of QoL > 1 and “visible blood in urine” = 0	27 (79.41%)	7 (20.59%)
G) summary score of 4 FDA symptoms ≤ 4, no item > 1 and “visible blood in urine” = 0	28 (82.35%)	6 (17.65%)
H) summary score of 3 EMA symptoms ≤ 3, no item > 1 and “visible blood in urine” = 0	28 (82.35%)	6 (17.65%)

As a next step, we tested the positive achievement of “clinical cure” rates by the eight thresholds in association to outcome using the “Dynamics” domain considering “clinical cure” as (1) resolution of symptoms (feeling normal) and (2) feeling much better. Due to lack of sufficient cases at the very early visit (< 2 days between “diagnostic” and “follow-up” evaluations), we decided to remove these 23 cases from this kind of evaluation. Thus, 213 cases of the total were included in further analysis.

The ROC-curve analysis of the different thresholds concerning the overall clinical outcome as reported by the patients in the “Dynamics” domain, demonstrated that the comparatively largest AUC (average [95% CI]) was noted for the threshold category B (Summary score of the “Typical” domain up to 4 AND no visible blood in the urine) at the “Early evaluation” (0.83 [0.75; 0.91]). It was as well comparatively larger for other terms of evaluation: 0.78 [0.64; 0.92] and 0.83 [0.65; 1.00] for the “End of treatment” and “Test of cure” evaluations. However, the differences were not statistically significant when compared either with other thresholds or other terms of evaluation (Suppl. Figure 1 a–c, Suppl. Table 1).

Highest value of sensitivity (average [95% CI]) was revealed for the threshold “A” (0.91 [0.85; 0.95]), the highest value of specificity was revealed for the threshold “F” (0.77 [0.65; 0.86]). The most optimal balance between sensitivity and specificity, positive and negative likelihood ratios, also highest Youden index and strongest correlation with the positive outcome (“Success”, according to the “Dynamics” domain of the “follow-up part B” form of the ACSS) was found to be for the threshold “D” (Summary score of the “Typical” domain up to 4 AND no “Typical” item > 1 in the absence of the visible blood in the urine): sensitivity (0.88 [0.81–0.92]) and specificity (0.74 [0.62–0.84]) (Suppl. Table 1).

## Discussion

Since a PROM is any report of the status of a patient’s health condition that comes directly from the patient, without interpretation of the patient’s response by a clinician or anyone else, the ACSS questionnaire could be such an instrument for female patients with AC. Besides the “Differential” and “Additional” domains (see above), the ACSS contains three different domains (Typical, QoL, Dynamics), which could be used alone or in combinations for this purpose. In the “Typical” domain, the patient is asked about six symptoms/signs, which she has already scored before, at visit 1, the diagnosis of AC was established. Although the symptoms asked for are usually considered typical for AC, none of the symptoms/signs can, however, be considered exclusive for AC. In earlier studies, it could be demonstrated, that the same symptoms in a mild form do not very well differentiate between patients with AC and controls without AC [12, 17]. Therefore, scoring of the symptoms is necessary to increase the diagnostic value of the so-called “typical” symptoms. The same applies for outcome criteria if symptoms are used for PROM, because the complete elimination of all symptoms cannot always be expected in all patients, although considered clinically cured. By scoring the severity of the symptoms, the threshold of most suitable reduction of symptoms needs to be analysed carefully below which a patient may be considered clinically cured. Therefore, scoring the severity of the symptoms also becomes relevant for PROM.

Although reports of patients concerning symptoms can only be subjective by definition, by answering the same, in the meantime, familiar questionnaire at any follow-up visit, one can at least expect that by scoring the symptoms not only the presence or absence, but also the increasing or decreasing severity of each symptom reported by the patient can be considered as a quasi-objective measure. Nevertheless, the amount of the reported change may still be subjective. Therefore, we do not consider a certain total summary score as a threshold to define “clinical cure”, but rather postulate that the symptoms do not exceed a severity of more than mild. Visible blood in urine, however, should become always absent, because persistent visible blood in urine would need further diagnostic steps to exclude serious pathologies, such as bladder cancer.

Besides symptom severity, the patient can also be asked about symptom discomfort (bothersomeness) and impact on daily and social activities (QoL domain) as considered necessary for PRO measures by Holm et al. [27]. Considering the QoL domain in addition, the results are closely related to the results using the symptom scoring system alone, but one gets the impression that for some patients, adjustment of their QoL takes somewhat longer than their awareness of symptom severity reduction.

Finally, in the ACSS, the patient is asked about her overall clinical assessment (“Dynamics” domain), which again considers more a relative change as compared to the situation before the AC has occurred (normal, baseline status) and compared to the situation when the diagnosis was established and any therapeutic intervention has started. The intention to correlate the overall patient’s clinical assessment with the reduction of the severity of typical symptoms was, however, not convincing. Unfortunately, we could not test the overall clinical assessments proposed in the draft guidelines by FDA and EMA [16, 17]. According to the draft EMA guidelines, the clinical outcome should be categorised as cure, failure or indeterminate. The cure may be defined as (1) complete resolution of clinical signs and symptoms and/or (2) sufficient improvement or return to baseline status such that no further antibacterial therapy is required for the index infection. According to the draft FDA guidelines, “clinical response” is defined as resolution of the symptoms of uUTI (dysuria, urinary frequency, urinary urgency, suprapubic pain) present at trial entry (and no new symptoms). Using both definitions, one probably will face the same problems, how patients consider “sufficient improvement” (EMA) or “resolution of symptoms” (FDA).

Considering these three different measures (symptoms, discomfort (bothersomeness) and impact on QoL, patient’s overall clinical assessment), it may be difficult to agree on the best PROM instrument for defining “clinical cure”. Using the ACSS for systematic reasons, we suggest the following two thresholds as the most appropriate for a suitable PROM instrument depending on the requirement not to include or to include QoL issues as strongly requested by Holm et al. [27]: (1) a summary score of the “Typical” domain up to 5 with no item > 1 (mild) AND no visible blood in the urine (threshold C); and (2) a summary score of the “Typical” domain up to 5 AND no “Typical” item > 1 (mild) AND no visible blood in the urine AND no “QoL” item > 1 (threshold E). If the threshold including QoL is used (E), one should consider that obviously “QoL improvement” is stated by some patients later than a reduction of symptoms’ severity. Whereas at visit 4 (end of treatment), the discrepancy between threshold C and E still were six cases (in favour of C), at visit 5 (test of cure), the difference was reduced to only one case.

The study has, of course, several limitations. It was a non-interventional study. The final diagnosis and treatment of AC were established by the treating physician according to international and national guidelines and standards. Because of the non-interventional character of the study, the follow-up visits of the patients could also not be defined a priori, but only grouped according to meaningful time intervals representing very early (< 2 days) and early (2–4 days) follow-up visits, end-of-treatment (5–9 days) and test-of-cure visits (> 10 days). Although all patients during the different follow-up categories were part of the cohort at visit 1 (diagnostics), the amount of cohorts during the different follow-up visits may also have differed very much between follow-up visits. Within a follow-up visit category, however, all parameters calculated referred to the same group of patients analysed at the beginning (diagnostic visit) and thus, were comparable.

In summary, the ACSS questionnaire was originally developed for clinical diagnostics and therapeutic outcome in female patients with acute uncomplicated cystitis (AC). During development, patients were interviewed, how they describe best the so-called typical symptoms of AC and their severity during an acute episode of AC and when they felt cured or improved after treatment, which was compared with controls without AC. In addition, the patients and the controls were asked about the impact on their quality of life according to three aspects (bothersomeness of symptoms, impact on daily life and work, impact on social life) and for their own overall clinical assessment after treatment. Therefore, the ACSS questionnaire can also be used as a PROM instrument, because patients were involved in the development, by focus groups and interviews to capture the breadth of symptoms and experiences associated with this particular disease, as requested by Rothrock et al. [28].

Nevertheless, it would be helpful to test the thresholds suggested in the current study to define “clinical cure” additionally in a prospective study with better-defined follow-up visits of all patients included.

## Conclusions

Since non-antibiotic therapy has become an alternative approach to treat AC in women, suitable PRO measures are urgently needed. Although typical symptoms are mainly used for clinical diagnosis and outcome, these symptoms are not exclusively found in AC. Therefore, severity scoring of the symptoms is needed not only for diagnostics, but also for PRO measure to define “clinical cure” of any intervention. The presented data analysis demonstrated that the ACSS questionnaire has the potential to be used as a suitable instrument for PRO in well-designed prospective clinical studies.

## Electronic supplementary material

Below is the link to the electronic supplementary material.

Supplementary material 1 (DOCX 76 kb)

Supplementary material 2 (XLSX 42 kb)
