# Supplementary material for: Additional assessment of Acute Cystitis Symptom Score questionnaire for patient-reported outcome measure in female patients with acute uncomplicated cystitis: part II
Source: World J Urol. 2019 Sep 23;38(8):1977–88. doi: 10.1007/s00345-019-02948-8 (PMC7363736; doi:10.1007/s00345-019-02948-8)
Supplement: Supplementary file 1 — Supplementary material 1 (DOCX 76 kb) [file 345_2019_2948_MOESM1_ESM.docx]

Suppl. Figure 1. Normal Q-Q of analyzed variables

Supp. Figure 2. Histogram of distribution of analyzed variables

Suppl. Figure 3 a-c ROC curves at three different follow up visit categories (early, end of treatment, and test of cure for the eight thresholds (explanation see text) versus “Dynamics” domain of the ACSS.

Suppl. Figure 1. Normal Q-Q of analyzed variables


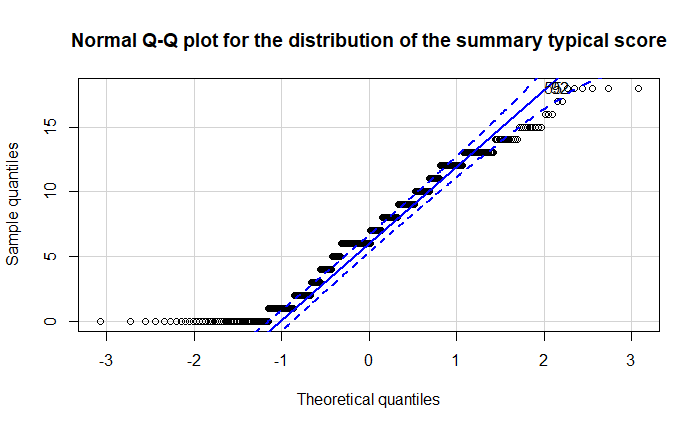


Supp. Figure 2. Histogram of distribution of analyzed variables


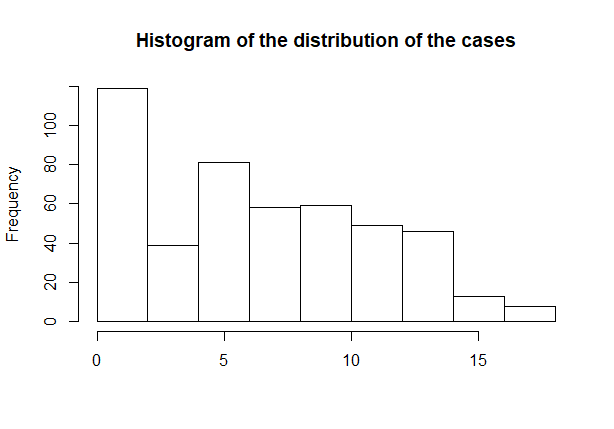


Suppl. Figure 3 a-c ROC curves at three different follow up visit categories (early, end of treatment, and test of cure for the eight thresholds (explanation see text) versus “Dynamics” domain of the ACSS.


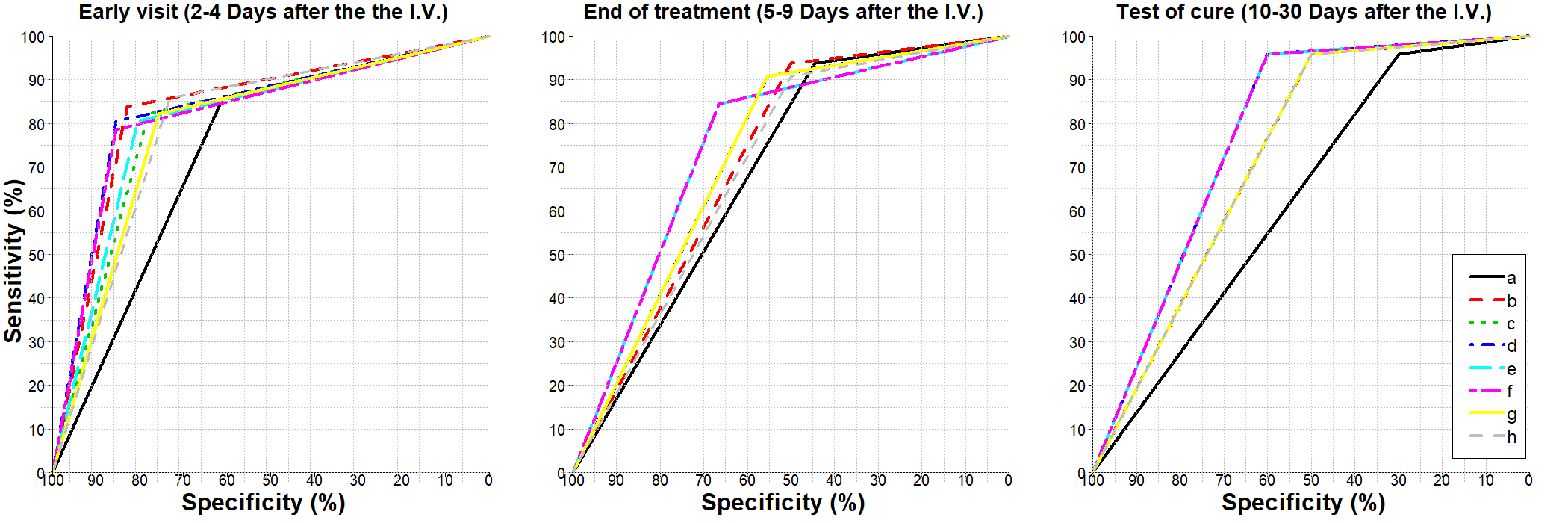


Legend: a) A summary score of the "Typical" domain up to 5 AND no visible blood in the urine

b) A summary score of the "Typical" domain up to 4 AND no visible blood in the urine

c) A summary score of the "Typical" domain up to 5 with no item >1 (mild) AND no visible blood in the urine

d) A summary score of the "Typical" domain up to 4 AND no "Typical" item >1 (mild) AND no visible blood in the urine

e) A summary score of the "Typical" domain up to 5 AND no "Typical" item >1 (mild) AND no visible blood in the urine AND no "QoL" item >1

f) A summary score of the "Typical" domain up to 4 AND no "Typical" item >1 AND no visible blood in the urine AND no "QoL" item >1

g) A summary score of the four FDA symptoms up to 4 AND no score >1 (mild) AND no visible blood in the urine

h) A summary score of the three EMA symptoms up to 3 AND no score >1 (mild) AND no visible blood in the urine
